# Effects of digital health interventions in women with high-risk pregnancies: a systematic review

**DOI:** 10.4069/whn.2024.12.06

**Published:** 2025-02-28

**Authors:** Sehee Kim, Mihyeon Park, Sukhee Ahn

**Affiliations:** 1 Department of Nursing, Pai Chai University, Daejeon, Korea; 2 Department of Nursing, Baekseok Culture University, Cheonan, Korea; 3 College of Nursing, Chungnam National University, Daejeon, Korea

**Keywords:** Digital health, High-risk pregnancy, Pregnancy complications

## Abstract

**Purpose:**

This systematic review evaluated the effectiveness of digital health interventions (DHIs) using technology-based strategies for women with pregnancy complications.

**Methods:**

Six databases were searched: MEDLINE, Embase, Cochrane Library, CINAHL, ProQuest, and Web of Science. The main concepts of interest were (1) target population: women with high-risk pregnancies; (2) health condition: pregnancy complications; (3) interventions: DHIs operationalized via mobile applications, the internet, and text messages; and (4) study design: randomized controlled trials (RCTs). The literature search was performed up to August 31, 2024.

**Results:**

The seven RCTs included in this review were published between 2016 and 2022. These studies originated from three countries: the United Kingdom, Switzerland, and the United States, and involved a total of 5,550 women. Two studies focused on gestational hypertension, four addressed preterm labor, and one dealt with preeclampsia. The interventions were delivered through phone applications or web platforms. While the primary outcomes did not show significant differences between the intervention and control groups across all studies, DHIs demonstrated meaningful secondary outcomes, including reductions in anxiety and stress among high-risk pregnant women.

**Conclusion:**

This review highlights the potential of DHIs to play a vital role in managing high-risk pregnancies. They facilitate early detection and risk prediction of pregnancy complications. Furthermore, digital health tools can markedly reduce stress and anxiety among high-risk pregnant women by providing psychological support. Overall, DHIs present a comprehensive approach to managing physical risks and improving mental well-being in women with high-risk pregnancies.

## Introduction

A high-risk pregnancy is defined as any pregnancy with a medical or obstetric condition that may adversely affect maternal or fetal outcomes and threaten the health or life of the mother or her fetus [1]. The complications of high-risk pregnancies include preeclampsia, placental abruption, placenta previa, premature birth, cervical incompetence, postpartum bleeding, amniotic fluid and membrane issues, and gestational diabetes mellitus. These conditions require close monitoring, and women experiencing such complications are often referred to specialized obstetric centers for management [2]. Although specific statistical data on the prevalence of all types of high-risk pregnancies are lacking, the incidence of high-risk complications has risen alongside the increasing average age of women at their first pregnancy [3]. A 2017 report from the Korea Centers for Disease Control and Prevention noted prevalence rates of 5.8% for gestational diabetes mellitus and 1.3% for preeclampsia. Additionally, other studies indicate that approximately 7.7% of births in 2018 were premature [2,4]. High-risk pregnancies account for 6% to 8% of all pregnancies in the United States and South Korea and are a significant concern due to their potential to lead to maternal death [5]. However, timely detection and proper management of high-risk complications can prevent up to 60% of maternal deaths [6,7]. Therefore, rigorous health monitoring during pregnancy is necessary to optimize outcomes for both the mother and the newborn in cases of high-risk pregnancies.

Various interventions have been implemented for different high-risk pregnancy diagnoses. One systematic review compared the effectiveness of these interventions in preventing preterm labor [8]. Another review determined that dietary and lifestyle changes could potentially lower the risk of preeclampsia [9]. Additionally, a meta-analysis indicated that physical activity may protect against the development of gestational diabetes mellitus [10]. However, the accessibility of these interventions is limited in scenarios such as the coronavirus disease 2019 (COVID-19) pandemic and is influenced by economic status, geographic location, and the availability of medical infrastructure for high-risk pregnancies.

Digital health interventions (DHIs) are increasingly utilized across various healthcare sectors. The use of mobile health or digital technologies offers a low-cost, easily accessible method to manage pregnancy complications. DHIs in preventive and personalized healthcare specifically target women with high-risk pregnancies [11]. These interventions provide automated and remote support for self-management, offering users the advantages of flexibility and convenient access to nursing interventions and feedback. In cases of high-risk pregnancy complications, DHIs not only enable women to self-monitor vital health indicators such as blood pressure [12] but also provide internet-based stress management to support psychological well-being during pregnancy [13]. DHIs are particularly promising for pregnant women experiencing preterm labor, who often face mobility restrictions [14,15]. The COVID-19 pandemic has increased the need to provide state-of-the-art interventions and monitoring as long-term support for women with high-risk pregnancies. Pregnant women regularly engage with digital health technologies and have shown a willingness to participate in web-supported perinatal interventions [16]. Previous reviews of high-risk pregnancies have mostly focused on in-person, hospital-based, pharmaceutical interventions [8], with few reviews examining DHIs for women with high-risk pregnancies. This systematic review aims to evaluate the effectiveness of DHIs, which are technology-based strategies designed for women experiencing pregnancy complications.

## Methods

### Ethics statement

As this review analyzed existing literature, it was exempt from institutional review board approval.

### Study design

The review adheres to the Preferred Reporting Items for Systematic Reviews and Meta--analyses (PRISMA) 2020 guidelines. The study was registered in the International Prospective Register of Systematic Reviews (PROSPERO, No. CRD42022312896) on November 28, 2022.

### Literature search methodology and data sources

**Figure 1.**
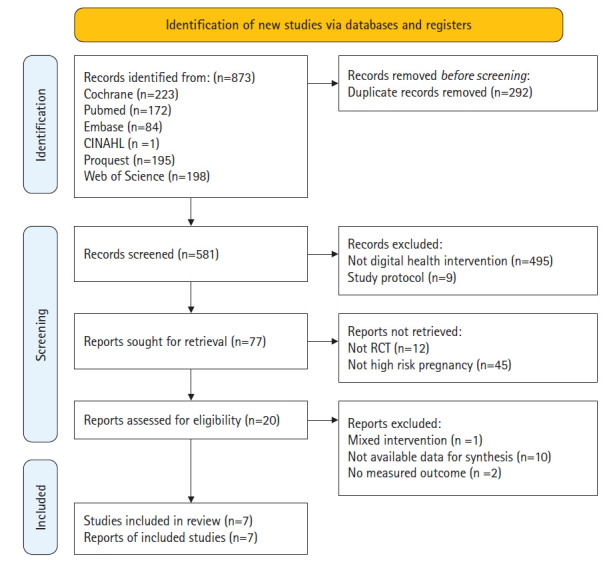
PRISMA 2020 flow chart for the literature review. RCT, randomized controlled trial.

Two authors conducted independent electronic searches to identify relevant studies, limiting their scope to English articles published in peer-reviewed journals. They searched six databases: MEDLINE, EMBASE, Cochrane Library, CINAHL, ProQuest, and Web of Science. The review focused on four main concepts: (1) target population, specifically women with high-risk pregnancies; (2) health condition, specifically pregnancy complications; (3) intervention, specifically DHI operationalized through mobile applications, the internet, and text messaging; and (4) study design, specifically randomized controlled trials. The review questions were formulated using the PICO-SD (Participants, Intervention, Comparison, Outcome, and Study Design) framework to identify studies that applied DHIs to women with high-risk pregnancies. The search strategy included both keyword and subject heading searches using MeSH and Emtree. The search terms used were: (“eclampsia” OR “preeclampsia” OR “gestational hypertension” OR “premature labor” OR “premature obstetric labor” OR “placenta previa” OR “incompetent cervix” OR “pregnancy complications”) AND (“app” OR “cell phone” OR “digital health” OR “ehealth” OR “email” OR “internet” OR “mhealth” OR “mobile” OR “online” OR “smart phone” OR “sms” OR “technology” OR “text message” OR “web”) AND (“randomized controlled” OR “pilot study”) (Appendix 1). Additionally, manual searches were conducted using Google Scholar to locate relevant studies. There were no restrictions on the publication dates of the studies, and the literature search was conducted up to August 31, 2024. Figure 1 presents a flow chart of the search process for article inclusion in this study.

### Inclusion and exclusion criteria

Studies were selected if they met the following criteria:

• Participants: Pregnant women diagnosed with preterm labor, preeclampsia, gestational hypertension, placenta previa, cervix incompetence, or any other condition classified as high-risk pregnancy.

• Intervention: Interventions using all types of digital tools were selected if they were used to manage health conditions during pregnancy, including smartphone applications, internet, and text messages.

• Comparison: Standard of care.

• Outcomes: Maternal/fetal/neonatal outcomes, adverse events, and psychological outcomes.

• Study design: Only randomized controlled trials involving women with high-risk pregnancies were included.

The following types of studies were excluded from this analysis: survey studies, qualitative studies, literature reviews, and studies only reported as abstracts at conferences. Gestational diabetes mellitus was also excluded from consideration, as it has been extensively studied and systematic reviews of DHIs for this condition have already been published [17,18].

### Study selection

Relevant studies were first identified independently by two authors (SK, MP). Duplicate articles were removed from the list of studies using EndNote software. The authors thoroughly reviewed the title and abstract of each study to check whether the study met the inclusion criteria. Those two authors independently cross-checked chosen studies that met the criteria. The same two authors then evaluated the full texts of potentially relevant studies. Any disagreements on study selection between the authors were resolved through discussion, and the third author (SA) made the final decisions on the inclusion of those studies.

### Data extraction and synthesis

Data extraction was performed by two authors (SK, MP) using a predefined form in Google Sheets to ensure consistency and accuracy. The extracted data encompassed study characteristics (authors, publication year, country), participant characteristics (age range, specific high-risk pregnancy conditions, sample size), details of interventions (technologies, methods), outcomes (primary and secondary), and results. Studies were categorized according to the type of pregnancy complication and the intervention used. A detailed summary of the key findings from each study was prepared, with a focus on the outcomes. The effectiveness of DHIs was evaluated. The characteristics of these DHIs were closely examined, including their main focus, frequency and duration of use, and any reported challenges or facilitators of implementation. This analysis aimed to identify common features of effective interventions. The context in which each study was conducted was analyzed to understand the influence of external factors such as study area and participants' demographics. The synthesis also highlighted gaps in the current literature, and recommendations for future research were formulated based on these gaps.

### Quality assessment

We assessed the methodological quality of all included studies using version 2 of the Risk of Bias (RoB 2) tool from the Cochrane Collaboration, which contains five domains of evaluation [19]. Two authors independently assessed the quality of each study. The quality assessment was conducted through consensus between the two authors, with a third author available to resolve any disagreements.

## Results

### Study characteristics

#### General and obstetric characteristics of included studies

The initial search yielded 873 studies, from which 292 duplicates were removed. After reviewing the remaining titles and abstracts, 20 studies were selected. Ultimately, seven studies [13-15,20-23] were included in this review. These studies, published between 2016 and 2022, originated from three countries: the United Kingdom, Switzerland, and the United States, and involved a total of 5,550 women. Two of the studies focused on gestational hypertension [20,21], four targeted preterm labor [13-15,23], and one dealt with preeclampsia [22]. All enrolled participants were in their second or third trimester (Table 1).

#### Type of technology

All studies used one type of technology for the intervention: either a phone application or the web (Table 2). The participants in five studies self-reported their conditions via an application [15,20-23]. Two studies used an internet-based program implemented as a step-by-step module [13,14]. Pregnant women diagnosed with gestational hypertension or preeclampsia were trained to monitor their blood pressure daily at home using a validated automated electronic sphygmomanometer. They transmitted their readings through the Microlife WatchBP Home application (Microlife Corp. Taipei, Taiwan) supported by a web-based data entry system. The QUantitative Innovation in Predicting Preterm birth (QUiPP) application (developed by Women's Health Academic Centre, King's College London) was employed to manage women experiencing preterm labor, following face-to-face training [15,23]. Women with preterm labor were instructed to attend an internet-based cognitive behavioral stress management (IB-CBSM) program with 6 weeks of online sessions and received feedback after each session [13,14].

#### The main focus of the intervention

Three studies focused on managing gestational hypertension via self-monitored blood pressure during pregnancy [20-22]. Two studies aimed to reduce levels of anxiety and decisional conflict in participants with preterm labor by allowing clinicians to communicate with them in an easy-to-understand risk-score format in smartphone apps [15,23]. Two studies investigated whether IB-CBSM interventions reduced stress/anxiety and led to better birth outcomes [13,14].

#### Intervention outcomes

The studies on gestational hypertension primarily relied on daily self-reported blood pressure measurements. Additionally, they monitored maternal, fetal, and neonatal outcomes, adverse events, and psychological factors as secondary outcomes [20-22]. The QUiPP studies utilized a five-page questionnaire booklet to gather demographic and medical information, symptoms, clinical assessments, anxiety scores (Visual Analogue Scale for Anxiety), and decisional conflict scores [15,23]. One IB-CBSM study assessed both physiological indicators, such as birth outcomes and salivary cortisol levels, and psychological indicators, including anxiety, stress, and depression. In contrast, another study focused solely on psychological outcomes, measuring stress, anxiety, satisfaction, and working alliance through self-reported tools [13,14].

### Quality assessment of selected studies

**Figure 2.**
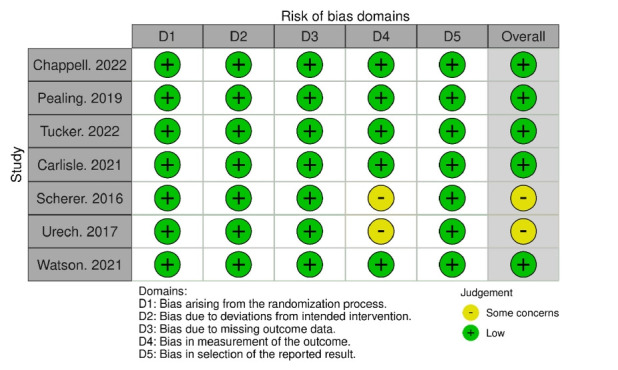
Assessment of the risk of bias of the included studies.

The findings of the quality assessment are illustrated in Figure 2. We conducted quality assessments of seven randomized controlled trials using the RoB 2 tool. The bias risk was assessed as low for five of the studies, while two were categorized as having some concerns. These two studies fell into domain 4 (bias in outcome measurement) because participants were aware of their group assignments. A self-reported assessment tool was employed to gauge psychological outcomes, although another study also gathered objective data, including birth outcomes and cortisol levels. The classification of “some concerns” in domain four stemmed from the possibility that knowing the assigned intervention could influence the outcomes reported by participants. However, the impact of this knowledge remains uncertain, as the descriptions of the interventions in the studies were not detailed.

### Effectiveness of digital health interventions

The expected outcomes of the DHIs included lowering blood pressure in studies on gestational hypertension and enhancing early risk detection of preterm labor to improve pregnancy outcomes or decrease the psychological burden during pregnancy in the preterm labor studies. While the primary outcomes did not show significant differences between the intervention and control groups across all studies, the use of digital technology interventions could still offer physiological and psychological benefits for high-risk pregnancies, as indicated by meaningful secondary outcomes in some studies.

#### Mobile-phone-based intervention: Microlife WatchBP Home application

Three gestational hypertension studies used the Microlife WatchBP Home smartphone application as the intervention [20-22]. There was a high level of persistence in the self-monitoring intervention, with 76% of participants in Tucker et al. [22] and over 80% in Pealing et al. [21] consistently providing home blood pressure readings. The mean systolic blood pressure, the primary outcome, showed no significant differences between the intervention and usual care groups in both the chronic and gestational hypertension categories across all three studies. All three studies reported serious adverse events that were unrelated to the intervention in either the mothers or the infants. Additionally, there were no significant differences in adverse events or maternal/infant outcomes between the intervention and usual care groups. No differences were observed in the mean scores for quality of life and anxiety after the intervention [20-22]. However, participant preferences suggested that home blood pressure monitoring was associated with reduced anxiety [21]. The intervention groups in two of the studies also demonstrated significantly improved scores on the Brief Illness Perception Questionnaire [20,22].

#### Mobile phone-based intervention: QUiPP application

Two studies on preterm labor provided interventions through mobile phones using the QUiPP application to support women at risk [15,23]. There was a significant reduction in anxiety scores from before to after the intervention. However, no significant reductions in anxiety and decisional conflict were observed in women who knew that the QUiPP application was part of the intervention [23]. Although the management of preterm labor did not differ significantly between the intervention and control sites, unnecessary admissions and discharges could have been reduced at the intervention sites if the QUiPP risk assessment had been properly implemented at the control sites as per the protocol [15]. In cases of adverse outcomes in women with preterm labor, those at the intervention sites more frequently received the management recommended by United Kingdom national guidelines [15]. The QUiPP application, along with its receiver operating characteristic, accurately predicts delivery within 7 days [15].

#### Internet-based (web-based) intervention: IB-CBSM program

Two studies on preterm labor utilized the IB-CBSM program to support psychological well-being during pregnancy [13,14]. Despite significant reductions in stress and anxiety levels from pre- to posttreatment in both groups, no differences were observed between the groups [13]. Similarly, Urech et al. [14] found no differences in birth outcomes or psychological well-being (depression, anxiety, and stress) between the intervention and control groups, even though psychological well-being improved following both interventions. The intervention group demonstrated significantly higher adherence to the study protocol than the control group. The cortisol awakening response, used as a physiological indicator, showed no significant variations across the groups or over time [14]. Women in the intervention group reported significantly higher satisfaction and Working Alliance Inventory (WAI) scores than those in the control group. Additionally, significant correlations were observed between the Task and Goal subscales of the WAI and levels of stress/anxiety [13].

## Discussion

This systematic review aimed to evaluate the effectiveness of DHIs that employ technology-based strategies for women experiencing high-risk pregnancies. Although the primary outcomes showed no significant differences between the intervention and control groups across the studies, DHIs may provide physiological and psychological benefits for high-risk pregnancies, as suggested by several studies with significant secondary outcomes.

According to studies on gestational hypertension, home blood pressure monitoring did not significantly improve blood pressure control compared to usual clinic care. The similar levels of blood pressure control between home and clinic monitoring suggest that monitoring blood pressure at home is as reliable as in the clinic. This interpretation is supported by a previous systematic review, which found no systematic difference between self-monitored and clinic blood pressure readings. This indicates that the appropriate treatment and diagnostic thresholds for self-monitoring should be equivalent to those used in the clinic [24]. Chappell et al. [20] reported that 25% of pregnant participants who had normal blood pressure readings at home but elevated readings in the clinic were experiencing white-coat syndrome. The studies reviewed demonstrated that by avoiding the white-coat effect during pregnancy, DHIs could benefit women with gestational hypertension by allowing them to detect hypertension at home. Furthermore, the absence of significant differences in adverse events and maternal outcomes between the intervention and usual care groups supports the safety of home blood pressure monitoring compared to clinic monitoring. Home blood pressure monitoring can also decrease the frequency of clinic visits and unnecessary hospital admissions for women with gestational hypertension. This not only reduces the financial burden but also provides these women with more rest in the comfort of their home environment, which is crucial for managing high-risk pregnancies.

QUiPP interventions primarily involved healthcare providers who utilized the tool to triage preterm labor. At the control sites, clinicians integrated elements of QUiPP rather than adhering strictly to national guidelines, a factor that may explain the observed lack of impact on reducing unnecessary admissions for preterm labor. Additionally, QUiPP proved highly effective in predicting delivery within 7 days. Notably, a higher number of women experiencing adverse outcomes at the control sites did not receive the recommended management regimen. This finding aligns with those of a previous study [25]. These results provide strong evidence that QUiPP could serve as a valuable guideline for clinical decision-making in cases of preterm labor by accurately forecasting imminent delivery, thereby assisting clinicians and pregnant women in determining the most suitable management approach and timing.

Home blood pressure monitoring was associated with reduced anxiety and improved illness perceptions and was preferred in three studies [20-22]. The QUiPP studies also observed reductions in anxiety and decisional conflict among women with preterm labor, although the mean difference between groups was not statistically significant. In the IB-CBSM studies that provided stress management interventions to women with preterm labor, the intervention group reported in Scherer et al. [13] showed higher satisfaction and working alliance, which significantly correlated with reduced stress/anxiety. Meanwhile, Urech et al. [14] noted reductions in stress/anxiety from pre- to post-intervention in both the intervention and control groups. Psychological well-being is crucial in managing high-risk pregnancies, as psychological factors such as anxiety, stress, and depression are positively associated with increased risks in these pregnancies [26-28]. Although the studies reviewed may not have provided conclusive evidence that DHIs reduce stress or anxiety and enhance psychological well-being, the potential for DHIs to support perinatal mental health care is rapidly increasing [29].

Birth outcomes did not differ significantly between the intervention and control groups in most of the studies, indicating similar levels of infant safety regardless of whether women with high-risk pregnancies received DHIs or usual clinical care.

Intervention persistence/adherence was reported in three studies [14,21,22]. Approximately 80% of the participants maintained adherence to the intervention from enrollment through to delivery, which another study recognized as good adherence [30]. Participants in the IB-CBSM also demonstrated significantly higher adherence compared to those in the control group [21]. High adherence to interventions is essential for achieving desired outcomes, especially since DHIs demand greater participant engagement than those providing human support [31]. These studies, which reported high adherence rates, suggest that DHIs are well-received or even preferred by women with high-risk pregnancies, emphasizing that adherence is crucial for enhancing the effectiveness of interventions.

Information technology usability and accessibility are crucial for the success of DHIs. Programs like the IB-CBSM software require users to be comfortable with computers and web-based platforms, which can significantly influence outcomes, particularly psychological ones. Mobile applications such as Microlife WatchBP Home and QUiPP, used in interventions, must be user-friendly and allow easy access and log in at any time. In this review, we were unable to assess technical issues associated with DHIs, as no problems were reported during the trials. The use of blood pressure monitoring devices may encounter barriers, as identified in a previous study. These include lack of supportive supervision, issues with charging the device, misleading displays, and damage to device components [32]. Another potential barrier is poor communication between clinicians and pregnant women, which is crucial for the effective use of QUiPP. To minimize decisional conflict and anxiety among women experiencing preterm labor, it is essential for them to engage in active discussions with clinicians about QUiPP scores during decision-making processes. Since clinicians are the primary users of the QUiPP app, fostering effective communication is a key challenge in designing DHIs.

This review had some limitations. Since DHIs are relatively new innovations and only randomized controlled trials were selected for this review, only two diagnoses were identified in the seven studies. Regarding DHI methods, the review focused on two phone applications and one web-based program, indicating a lack of diversity in the types of DHIs evaluated. Additionally, there was variability in the outcome measures and the timing of interventions during the pregnancies, which precluded the performance of a meta-analysis to compare effect sizes.

High-quality investigations of digital health necessitate multidisciplinary collaboration from various fields including medicine, science, psychology, education, and engineering [33]. Therefore, conducting and evaluating DHIs to support women with high-risk pregnancies requires insights from a broad range of disciplines, and may benefit from including pregnant women as part of the research team. Another study recommends that designers of DHIs aimed at supporting pregnant women with anxiety should include web-based interactions with therapists, healthcare professionals, and peers [34]. Future research on DHIs should ensure a minimal level of online interaction with healthcare providers to provide reassurance to women experiencing high-risk pregnancies and elevated levels of psychological stress. The current review did not address certain barriers or challenges faced during implementation, such as cost analyses, ease of use, device reliability, data security, and communication with healthcare providers. Future studies should explore and quantify how these and other factors influence effectiveness.

In conclusion, this review highlights the potential of using DHIs to support and monitor women with high-risk pregnancies, to facilitate the early detection or risk predictions of pregnancy complications, and to attenuate stress/anxiety. DHIs ensure patient safety and are as effective as clinic care, thereby reducing the need for frequent clinic visits. This reduction may allow for increased rest in the home environment, a fundamental principle in the management of high-risk pregnancies.

## Figures and Tables

**Table 1.** Study characteristics of the selected RCTs (N=7)

High-risk pregnancies	Study	Year	Country	Participants		Gestational weeks	Study outcomes	Main findings
				Intervention	Control			
Gestational hypertension	Chappell et al. [20]	2022	UK	430	420	20–37	1. Primary outcome: BP	1. Primary outcome
							2. Secondary outcomes:	No significant differences between the SMBP group and the usual care group
							1) Psychological: QoL, anxiety, illness perception	2. Secondary outcomes
							2) Maternal and neonatal: serious maternal complications, perinatal outcomes, adverse events	No significant differences in gestational age at birth or infant outcomes
							3) Others: medication adherence	No significant differences in adverse events between the groups
								No significant differences in medication adherence between the groups
								The SMBP group had a lower proportion of spontaneous labor onset than the usual care group (OR, 0.52; 95% CI, 0.29–0.92)
								The SMBP group had higher scores on the modified Brief Illness Perception Questionnaire than the usual care group (*p*<.001).
Gestational hypertension	Pealing et al. [21]	2019	UK	49	23	20–37	1. Primary outcome: BP	1. Primary outcome
							2. Secondary outcomes:	No significant differences between the SMBP group and the usual care group
							1) Psychological: QoL, anxiety, medication beliefs	2. Secondary outcomes
							2) Maternal and neonatal: serious maternal complications, perinatal outcomes, adverse events	No significant differences in adverse events, QoL, or anxiety between the groups, although the SMBP group reported lower anxiety and higher control with home monitoring
							3) Others: medication adherence, BP monitoring preference (problem score)	Lower problem score for the SMBP group (MD, –0.7; 95% CI, –0.9 to –0.4)
								High adherence in the SMBP group
Gestational hypertension/preeclampsia	Tucker et al. [22]	2022	UK	1,223	1,218	16–24	1. Primary outcome: BP	1. Primary outcome
							2. Secondary outcomes:	No significant difference between the SMBP group and the usual care group.
							1) Psychological: QoL, anxiety, illness perception	2. Secondary outcomes
							2) Maternal and neonatal: serious maternal complications, perinatal outcomes, adverse events	No significant differences in the incidence of spontaneous onset of labor, anxiety, and QoL between the groups
							3) Others: medication adherence	The SMBP group had higher scores on the modified Brief Illness Perception Questionnaire than the usual care group (*p*<.001)
								No significant differences in perinatal outcomes and adverse events between the groups.
								High adherence in the SMBP group
Preterm labor	Carlisle et al. [23]	2021	UK	81	121	23–34	1. Primary outcomes: anxiety, decisional conflicts	1. Primary outcomes
								Anxiety levels significantly decreased for women in QUiPP sites, but not statistically significant (t=1.13, *p*=.26)
								Awareness of using the QUiPP application was associated with lower decisional conflict but without statistical significance (*p*=.09)
Preterm labor	Scherer et al. [13]	2016	Switzerland	51	42	18–32	1. Primary outcomes: stress, anxiety, program satisfaction	1. Primary outcomes
							2. Secondary outcome: working alliance	Significant reductions in stress and anxiety levels for both intervention and control groups (*p*<.001), but no significant differences between the groups
								The intervention group scored significantly higher on patient satisfaction (U=165, *p*<.001)
								2. Secondary outcome
								The intervention group scored significantly higher on working alliance (t=2.476, *p*=.016)
Preterm labor	Urech et al. [14]	2017	Switzerland	50	43	18–32	1. Primary outcomes: anxiety, stress, depression, salivary cortisol level, birth outcome	No significant difference in birth outcomes between the IB-CBSM group and the control group.
							2. Secondary outcome: adherence to the program	Significant improvements in anxiety, depression, and stress over time for both groups (*p*<.001), but no significant interaction effects between group and time
								No significant difference in cortisol awakening responses between groups and time
								The IB-CBSM group had significantly higher adherence to the program (*p*<.001)
Preterm labor	Watson et al. [15]	2021	USA	724	1,075	23–34	1. Primary outcome: unnecessary management	1. Primary outcome
							2. Secondary outcomes: maternal and neonatal adverse outcomes, process measures	No significant difference in the unnecessary management of TPTL between intervention sites and control sites
								Unnecessary admissions and discharges would have been reduced in the intervention sites if the QUiPP was used as per protocol (7.4% vs. 9.9%) (OR, 0.72; 95% CI, 0.45–1.16)
								2. Secondary outcomes
								Maternal adverse outcomes occurred in four patients from the intervention sites and 12 from the control sites without recommended management
								QUiPP was highly predictive of delivery in 7 days (ROC curve, 0.90; 95% CI, 0.85–0.95)

BP: Blood pressure; CI: confidence interval; IB-CBSM: internet-based cognitive behavioral stress management; MD: mean difference; OR: odds ratio; QoL: quality of life; QUiPP: QUantitative Innovation in Predicting Preterm birth; RCT: randomized controlled trial; ROC: receiver operating characteristic; SMBP: self-monitoring blood pressure; TPTL: threatened preterm labor.

**Table 2.** Description of the digital health interventions (N=7)

Study	Year	Technology	Intervention title	Intervention component details	Intervention user	Intervention period	Intervention management	Intervention involvement
Chappell et al. [20]	2022	Application	BUMP 2 (using Microlife WatchBP Home)	BP monitoring daily	Study participants	Enrollment to 8 weeks postpartum	Clinician	BP reading
		Text messages		Taking 2 readings 1 minute apart, submitting the second reading to the application			Midwives	Text messages
				Getting weekly reminders, motivational messages				Audit and follow-up
Pealing et al. [21]	2019	Application	OPTIMUM-BP (using Microlife WatchBP Home)	BP monitoring daily	Study participants	Enrollment to 6 weeks postpartum	Clinical team	BP reading
		Text messages		Taking 2 readings 1 minute apart, submitting the second reading to the application				Study visits
				Attending up to three antenatal study visits at 20, 28, and 32 weeks of gestation, a 6-week postnatal face-to-face visit, or telephone study visit				
Tucker et al. [22]	2022	Application	BUMP 1 (using Microlife WatchBP Home)	BP monitoring 3 times per week	Study participants	Enrollment to postpartum visit	Midwives	BP reading
				Taking 2 readings, submitting a second reading to the application				Phone contact
Carlisle et al. [23]	2021	Application	QUiPP	After the initial face-to-face training, participants were instructed to use the application for 6 weeks without any further face-to-face contact. During this period, two researchers read the comments and wrote free-text annotations	Study participants	Overall study period: 36 weeks	Midwives	Training sessions
Scherer et al. [13]	2016	Internet	IB-CBSM	Six online sessions consisted of psychoeducational information, relaxation exercises, interactive exercises, activity diary, stress and problem-solving protocol	Study participants	6 weeks (1 session per week)	Psychologist	Regular written guidance
				Participants received feedback every week and had the opportunity to ask questions about pregnancy to midwives			Midwives	Weekly feedback
								Q&A
Urech et al. [14]	2017	Internet	IB-CBSM	Six online sessions consisted of psychoeducational information, relaxation training, interactive exercises, emotional training, cognitive training, problem solving and training of enjoyment, and psychoeducation and coping	Study participants	6 weeks (1 session per week)	Psychologist	Weekly feedback via email
				Participants received feedback every week				
				Participants had the opportunity to exchange with others online				
Watson et al. [15]	2021	Application	QUiPP	Receiving face-to-face training on the use of the QUiPP application	Clinician	Overall study period: 37 weeks	Trial team	Training the use of the QUiPP application
				The clinician assessed pregnant women and inputs info to the application				Additional support face-to-face or via email/telephone

BP: blood pressure; BUMP: blood pressure monitoring in pregnancy; IB-CBSM: internet-based cognitive behavioral stress management; QUiPP: QUantitative Innovation in Predicting Preterm birth.

## Appendix 1. Search strategy

**Table:**

Database	Search term	Literature
Cochrane	#1 (“eclampsia”):ti,ab,kw 3121	223
	#2 (gestational hypertension):ti,ab,kw 2190	
	#3 (“high risk pregnancy”):ti,ab,kw 687	
	#4 (“high-risk pregnancies”):ti,ab,kw 324	
	#5 (“incompetent cervix”):ti,ab,kw 14	
	#6 (“placenta praevia”):ti,ab,kw 57	
	#7 (“pre-eclampsia”):ti,ab,kw 2686	
	#8 (pregnancy complication):ti,ab,kw 4421	
	#9 (“premature labor”):ti,ab,kw 1739	
	#10 (“preterm labor”):ti,ab,kw 1702	
	#11 #1 OR #2 OR #3 OR #4 OR #5 OR #6 OR #7 OR #8 OR #9 OR #10 11625	
	#12 (“APP”):ti,ab,kw 11339	
	#13 (“cell phone”):ti,ab,kw 1815	
	#14 (digital health):ti,ab,kw 7318	
	#15 (ehealth):ti,ab,kw 1841	
	#16 (internet):ti,ab,kw 16692	
	#17 (mhealth):ti,ab,kw 2650	
	#18 (“online”):ti,ab,kw 28117	
	#19 (smart phone):ti,ab,kw 1476	
	#20 (text message):ti,ab,kw 3437	
	#21 (web based):ti,ab,kw 14242	
	#22 (“technologies”):ti,ab,kw 6617	
	#23 #12 OR #13 OR #14 OR #15 OR #16 OR #17 OR #18 OR #19 OR #20 OR #21 OR #22 70871	
	#24 (“randomized controlled study”):ti,ab,kw 30067	
	#25 (“randomized control trial”):ti,ab,kw 16966	
	#26 (“randomized-controlled trial”):ti,ab,kw 677389	
	#27 (“randomized clinical trial”):ti,ab,kw 71580	
	#28 (“pilot study”):ti,ab,kw 54418	
	#29 #24 OR #25 OR #26 OR #27 OR #28 764859	
	#30 #11 AND #23 AND #29 223	
Pubmed	(((“eclampsia”[Title/Abstract]) OR (“gestational hypertension”[Title/Abstract]) OR (“high risk pregnancy”[Title/Abstract]) OR (“high risk pregnancies”[Title/Abstract]) OR (“incompetent cervix”[Title/Abstract]) OR (“cervical incompetence”[Title/Abstract]) OR (“placenta previa”[Title/Abstract]) OR (“preeclampsia”[Title/Abstract]) OR (“pregnancy complication”[Title/Abstract]) OR (“premature labor”[Title/Abstract]) OR (“preterm labor”[Title/Abstract]) OR (“uterine cervical incompetence”[Title/Abstract])) AND ((“app”[Title/Abstract]) OR (“apps”[Title/Abstract]) OR (“cell phone”[Title/Abstract]) OR (“digital health”[Title/Abstract]) OR (“ehealth”[Title/Abstract]) OR (“e mail”[Title/Abstract]) OR (“internet”[Title/Abstract]) OR (“mhealth”[Title/Abstract]) OR (“mobile”[Title/Abstract]) OR (“online”[Title/Abstract]) OR (“smart phone”[Title/Abstract]) OR (“sms”[Title/Abstract]) OR (“technology”[Title/Abstract]) OR (“text message”[Title/Abstract]) OR (“web”[Title/Abstract])) AND ((“randomized controlled”[Title/Abstract]) OR (“pilot study”[Title/Abstract]) OR (“randomized control”[Title/Abstract])))	172
Embase	(eclampsia:ti,ab OR “gestational hypertension”:ti,ab OR “high risk pregnancy”:ti,ab OR “high risk pregnancies”:ti,ab OR “incompetent cervix”:ti,ab OR “cervical incompetence”:ti,ab OR “placenta previa”:ti,ab OR preeclampsia:ti,ab OR “pregnancy complication”:ti,ab OR “premature labor”:ti,ab OR “preterm labor”:ti,ab OR “uterine cervical incompetence”:ti,ab) AND (“app”:ti,ab OR “apps”:ti,ab OR “cell phone”:ti,ab OR “digital health”:ti,ab OR ehealth:ti,ab OR “e mail”:ti,ab OR “internet”:ti,ab OR “mhealth”:ti,ab OR “mobile”:ti,ab OR “online”:ti,ab OR “smart phone”:ti,ab OR “sms”:ti,ab OR “technology”:ti,ab OR “text message”:ti,ab OR “web”:ti,ab) AND (“randomized controlled”:ti,ab OR “pilot study”:ti,ab OR “randomized control”:ti,ab)	84
CINAHL	AB (eclampsia OR gestational hypertension OR high risk pregnancy OR high risk pregnancies OR incompetent cervix OR placenta previa OR preeclampsia OR pregnancy complication OR premature labor OR preterm labor OR uterine cervical incompetence) AND AB (app OR apps OR cell phone OR digital health OR ehealth OR e mail OR internet OR mhealth OR mobile OR online OR smart phone OR sms OR technology OR text message OR web) AND AB (randomized controlled OR pilot study OR randomized control)	1
Proquest	abstract(eclampsia OR gestational hypertension OR high risk pregnancy OR high risk pregnancies OR incompetent cervix OR placenta previa OR preeclampsia OR pregnancy complication OR premature labor OR preterm labor OR uterine cervical incompetence) AND abstract(app OR apps OR cell phone OR digital health OR ehealth OR e mail OR internet OR mhealth OR mobile OR online OR smart phone OR sms OR technology OR text message OR web) AND abstract(randomized controlled OR pilot study OR randomized control)	195
Web of Science	TS=((“eclampsia” OR “gestational hypertension” OR “high risk pregnancy” OR “high risk pregnancies” OR “incompetent cervix” OR “cervical incompetence” OR “placenta previa” OR “preeclampsia” OR “pregnancy complication” OR “premature labor” OR “preterm labor” OR “uterine cervical incompetence”) AND (“app” OR “apps” OR “cell phone” OR “digital health” OR “ehealth” OR “e mail” OR “internet” OR “mhealth” OR “mobile” OR “online” OR “smart phone” OR “sms” OR “technology” OR “text message” OR “web”) AND (“randomized controlled” OR “pilot study” OR “randomized control”))	198
